# High SVR12 With 8-Week Course of Direct-Acting Antivirals in Adolescents and Children With Chronic Hepatitis C: A Comprehensive Analysis

**DOI:** 10.3389/fmed.2021.608760

**Published:** 2021-06-08

**Authors:** Zuqiang Fu, Chen Dong, Zhijun Ge, Chunhui Wang, Yun Zhang, Chao Shen, Jun Li, Chuanlong Zhu, Yan Wang, Peng Huang, Ming Yue

**Affiliations:** ^1^Department of Epidemiology, School of Public Health, Nanjing Medical University, Nanjing, China; ^2^Eastern Theater Command Centers for Disease Control and Prevention, Institute of Epidemiology and Microbiology, Nanjing, China; ^3^Department of Epidemiology and Statistics, School of Public Health, Medical College of Soochow University, Suzhou, China; ^4^Department of Critical Care Medicine, The Affiliated Yixing Hospital of Jiangsu University, Yixing, China; ^5^Department of Infectious Diseases, The First Affiliated Hospital of Nanjing Medical University, Nanjing, China

**Keywords:** hepatitis C virus, direct-acting antivirals regimens, adolescents and children, sustained virological response, treatment duration

## Abstract

Direct-acting antiviral (DAA) treatment for 8 weeks has a sustained virological response rate in adults with chronic hepatitis C. We have conducted a systematic review and meta-analysis to compare the efficacy and safety of the 8-week vs. 12/24-week DAA treatment in adolescents and children with CHC. The PubMed, Web of Science, and Cochrane databases were searched for the relevant articles from January 1, 2017 to August 28, 2020 and further screened for literature reviews on April 1, 2021. Pool proportions with 95% CIs for SVR12 were summarized with fixed/random effects models using Freeman–Tukey double arcsine transformation. Subgroup analysis was used to explore the source of heterogeneity. Thirty-six relevant publications were identified. For adolescents aged 12–17 years old, the pooled SVR12 and AE rate were 99.4% (95% CI: 98.7–99.9) and 34.7% (95% CI: 31.9–37.6). No one discontinued treatment due to drug intolerance. In addition, the SVR12 adolescents treated for 12 and 8/24 weeks were 99.3% (95% CI: 98.4–99.9) and 100%, respectively. The pooled SVR12 rate, AEs, and SAEs for children younger than 12 years were 98.9% (95% CI: 97.3–99.8), 51.6% (95% CI: 47.0–56.2), and 1.1% (95% CI: 0.4–2.5), respectively. The most common AE was fatigue (28.4%). The SVR12 was 98.8% (95% CI: 97.1–99.8) and 100% for the pediatric patients treated for 12 weeks and 8/24 weeks, respectively. Taken together, DAAs are generally effective against CHC and well-tolerated by the adolescents and children. A treatment duration of 8 weeks is equally effective and safe as 12/24 weeks in this demographic group.

## Introduction

Hepatitis C is caused by hepatitis C virus (HCV) infection and afflicted 71.7 million people or 1% of the global population in 2015 (1, 2), of which 13.2 (11.5–21.2) million were children and adolescents aged 1–15 years (3). Only 1.76 million (13%) of the patients received treatment, and 86% (1.51 million) were treated with direct-acting antivirals (DAAs) (1, 2).

Vertical HCV infection is cleared spontaneously without treatment in 20% of the pediatric patients, while the remaining 80% develop chronic infection in the first 4 years of life that usually persists into adulthood (4–6). Early diagnosis and treatment at younger age can reduce the prevalence of chronic infection in adulthood, and therefore reduce the global burden of HCV (7). However, although 5.5 million people with chronic HCV have been treated so far, most of these patients are adults that received the less effective interferon-based regimens (2).

The Food and Drug Administration (FDA) approved supplemental administration of sofosbuvir (SOF) and a combination of sofosbuvir and ledipasvir (SOF+LDV) in April 2017 to treat HCV in adolescents aged 12–17 years (8). In addition, several single-arm clinical trials conducted in the last 2 years have shown that DAAs are highly effective in pediatric CHC patients aged 6–12 years (9, 10). However, most of these studies have only analyzed the efficacy of DAAs on specific pediatric patient populations, such as those infected with HCV genotype 4 (GT) (11, 12), or the treatment experienced (TE) or treatment-naïve (TN) patients (13). The efficacy of short-duration (8 weeks) DAA treatment in adolescents and children with HCV infection has not been summarized so far.

The aim of this study was to comprehensively evaluate the efficacy and safety of 8-week vs. 12/24-week DAA regimens in adolescents and children with HCV infection using data from published studies. Our findings provide valuable information for medical professionals and researchers.

## Materials and Methods

This systematic review and meta-analysis was conducted according to the preferred reporting items for systematic review and meta-analyses (PRISMA) statement (Supplementary Table 1) (14).

### Literature Search

**Figure 1:**
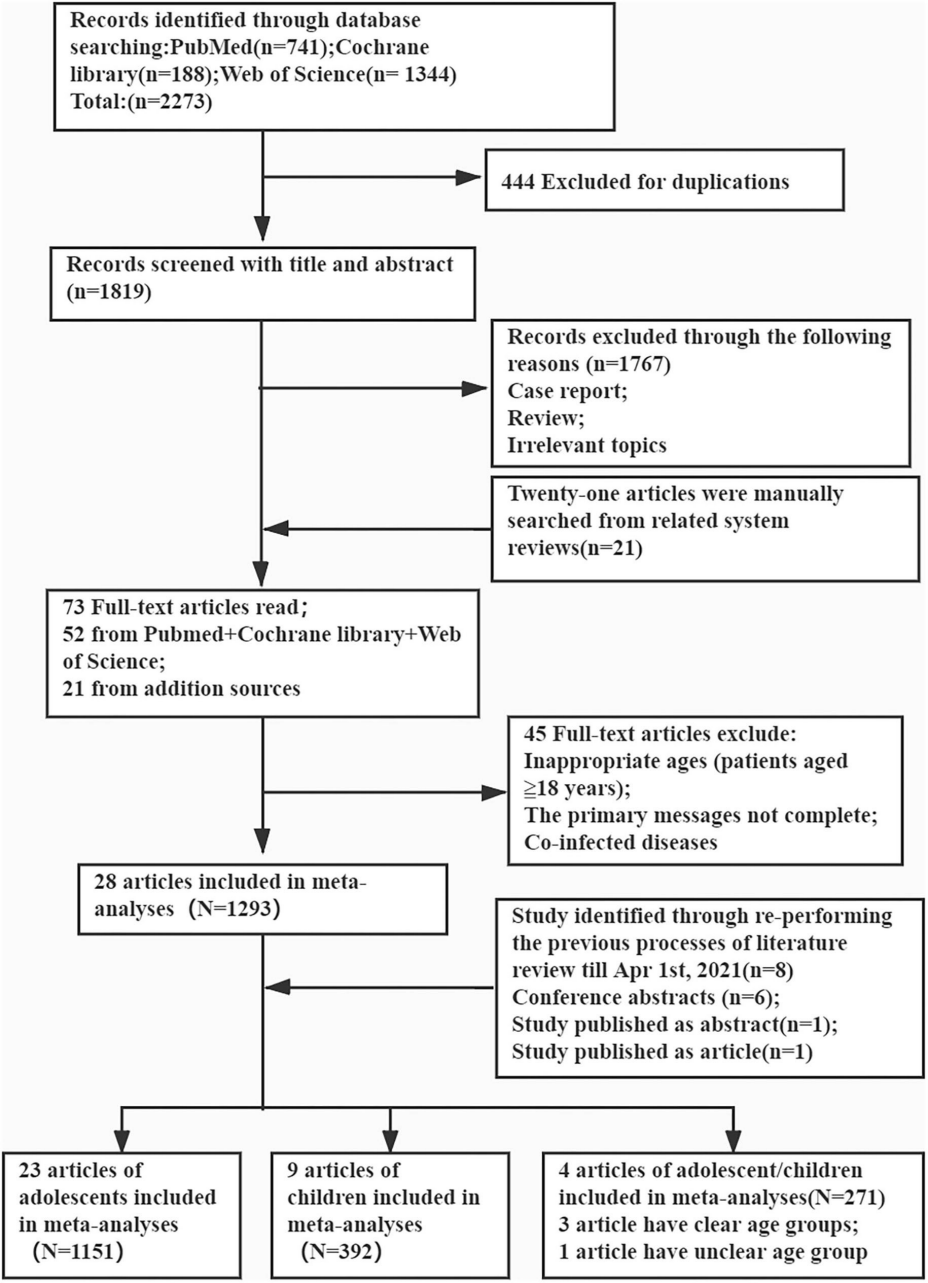
Preferred reporting items for the review flow diagram for identification of relevant studies.

PubMed, Cochrane Library, and Web of Science databases were searched for the relevant articles from January 1, 2017 to August 28, 2020. Literature reviews were searched on April 1, 2021. There were no restrictions on the year of publication and language. To avoid missing any study, several keywords were replaced with their synonyms. The following search terms were applied: “hepatitis C virus” (e.g., “HCV”; “CHC”; “hepatitis c”); “direct-acting antiviral” (e.g., “DAA”; “Sofosbuvir”; “Dasabuvir”; “Daclatasvir”; “Ledipasvir”; “Ombitasvir”; “Elbasvir”; “Velpatasvir”; “Boceprevir”; “Telaprevir”; “Simeprevir”; “Asunaprevir”; “Paritaprevir”; “Grazoprevir”); “pediatric” (e.g., “paediatr”; “pediatr”); and “children” (e.g., “child”; “teenager”; “kid”; “adolescent”; “youngster”; “juvenile”) (Supplementary Table 2). All types of studies were collated initially. The procedure is outlined in Figure 1.

### Inclusion and Exclusion Criteria

Studies that met the following criteria were included: (i) HCV infection (HCV RNA positive in blood) (8), (ii) adolescents (12–17 years) or pediatric (<12 years of age) patients, (iii) DAA treatment regimen, (iv) all HVC genotypes, (v) definite outcome variables (SVR12), (vi) TN or TE patients, and (vii) informed consent.

The exclusion criteria of the studies were as follows: (i) co-infection with HBV or HIV, (ii) evidence of HCC or other malignancy, (iii) history of solid organ or bone marrow transplantation, (iv) decompensated liver disease or chronic liver disease of a non-HCV etiology, (v) review, case report, or articles with >10 subjects, and (vi) not treated with any DAA-containing regimens.

### Study Selection

The duplicate studies were first eliminated using Endnote software, and the unrelated studies were excluded by browsing through the titles and abstracts. Studies with only adult subjects or lacking DAAs in the treatment regimens were excluded, and those reporting on the efficacy or safety of DAA treatment in children were retained. The bibliographies of the most recent relevant literature reviews were manually inspected to obtain additional articles. To avoid selection bias caused by one person, two reviewers (Mr. Fu and Miss Yue) evaluated all abstracts and selected the relevant studies for full-text reading. Any disagreement was resolved by consensus among all authors.

### Research Outcomes

The primary outcome was the efficacy of DAA regimens in adolescents and children, which was defined as the percentage of patients with SVR12 [HCV RNA < the lower limit of quantitation (LLOQ) at 12 weeks after cessation of therapy]. The SVR12 in this meta-analysis was the intention-to-treat (ITT) SVR12. The second outcome was the percentage of patients with adverse events (AEs) and serious AEs (SAEs). The AEs were defined as any unfavorable medical event reported by patients or any aberrations observed by the clinicians from the baseline laboratory indices after administration of the first dose until 30 days after the last dose. Common AEs included fatigue, nausea, and so on. The SAEs were defined as any event causing disability, congenital malformation, or death (8, 15–17). The worsening of laboratory test values from baseline was graded using the National Cancer Institute Common Terminology Criteria for Adverse Events (18). The safety of DAA regimen for HCV-infected patients was evaluated by the rate of drug-related AEs, SAEs, discontinuation, and laboratory abnormalities (19).

### Data Extraction and Quality Evaluation

All relevant data including SVR12 (the primary endpoints of interest), side effects, study characteristics (e.g., study author, publication date, study type, and study sites), patient characteristics at baseline (e.g., age, sex rate, genotype, and treatment regimen/duration), and possible factors that affect the outcomes of treatment were extracted from the articles. The study subjects were divided into the adolescents (12–17 years) and children (<12 years of age) groups.

All studies were assessed for methodological quality using the tool of Review Manager 5.2. The items of evaluation refer to a National Institutes of Health quality assessment tool: the tool for “Before-After (Pre-Post) Studies With No Control Group” (https://www.nhlbi.nih.gov/health-topics/study-quality-assessment-tools) (Supplementary Table 3). Each criterion was graded as “yes,” “no,” or “unclear,” which corresponded to “low bias risk,” “high bias risk,” and “unclear risk of bias,” respectively.

### Statistical Analyses

R x64-3.6.1 software (The R Foundation for Statistical Computing) was used for the meta-analysis. Pool proportions with 95% CIs for SVR were summarized with fixed effects models using Freeman–Tukey double arcsine transformation (20). Fixed/random effects models were used in all analyses, and statistical heterogeneity was calculated with the *I*^2^ method and subgroup differences using the Q-test. *I*^2^ was calculated as follows: *I*^2^ (%) = 100 × (Q − df)/Q, where Q is Cochrane's heterogeneity statistic and df indicates the degree of freedom. Negative values for *I*^2^ were set to zero, and an *I*^2^ ≥ 50% was considered to have substantial heterogeneity. Publication bias was analyzed by Funnel plots. *P* < 0.05 was considered statistically significant.

## Results

A total of 741, 1,344, and 188 studies were initially identified in the PubMed, Web of Science, and Cochrane Library databases, respectively, of which 444 duplicate articles were excluded. After screening the titles and abstracts, 1,767 articles were further excluded. After including 21 additional articles from manual search of the reference lists, a total of 73 papers were eligible for full-text screening, of which 45 were excluded for incomplete data and/or inappropriate age groups (patients aged ≥18 years) and 7 for patients with co-morbidities. Another eight articles were included after the later literature search. Finally, 36 articles were included for further review, except for one that included both children and adolescents (Figure 1).

### Studies and Patients' Characteristics

The main characteristics of the patients and studies are summarized in Table 1. A total of 28 studies were included, of which 18 were from Egypt, 7 from the United States, 4 from India, and 5 from multiple or other countries. All studies were observational, and 14 were multi-center studies. Except for three studies that did not specify the age groups, a total of 1,718 patients (1,253 adolescents and 465 children) were included in the studies, of which 792 were infected with HCV GT4, 545 with HCV GT1, 156 with GT3, 43 with HCV GT2, 1 with HCV GT5, and 213 with unknown GTs. Apart form 267 patients with unavailable treatment history, 1,216 were TN and 272 were TE. The majority of the patients (59%, 951/1,612) were males.

**Table 1:** Main characteristics of the studies and patients included in this review.

**References**	**Study design**	**Study sites**	**Genotype (n)**	**Treatment history (TN/TE)**	**Man** **(n, %)**	**Age (median)**	**Treatment regimen**	**Treatment duration (weeks)**	**Total** **(N)**	**Primary events, SVR (%, n/N)**
Rosenthal et al. (21)^**^	Multicenter, open-label	USA	G1 (26)	26/0	9 (35)	7.5 (3.0–11.0)	OBV/PTV/R+DSV+RBV	12	26	SVR12, 96.0 (25/26)
Fouad et al. (22)	Single-arm	Egypt	NA	36/8	28 (60.9)	13.5 (12–17)	SOF+LDV	12	46	SVR12, 100.0 (46/46)
Makhlouf et al. (23)	Open-label	Egypt	G4 (50)	6/44	36 (72.0)	13.6 (12–17)	SOF+LDV	12	50	SVR12, 100.0 (50/50)
Jonas et al. (18)	Open-label	USA	G1 (37), G2 (3), G3 (1), G4 (3)	36/8	21 (47.7)	14 (12–17)	G+P	8	44	SVR12, 100.0 (44/44)
Behairy et al. (13)	Single-arm	Egypt	G4 (30)	30/0	20 (66.7)	6.7 (4–10)	SOF+LDV	8	30	SVR12, 100.0 (30/30)
Schwarz et al. (24)	Multicenter, open-label	USA, UK, and Australia	G1 (33), G4 (1)	33/1	24 (71.0)	5 (3–5)	SOF+LDV	12	34	SVR12, 97.1 (33/34)
Kamal et al. (25)	Multicenter	Egypt	G4	22/0	19 (86.0)	4.8 (3–6)	SOF+LDV	8;12	22	SVR12, 100.0 (22/22)
Rosenthal et al. (26)^**^	Multicenter, open-label	USA	G2 (18), G3 (36)	53/1	14 (25.9)	6.5 (3–11)	SOF+RBV	12	54	SVR12, 98.2 (53/54)
Serranti et al. (27)	Multicenter open-label	Italian	G1 (14)	14/10	6 (42.9)	16.5 (12–7)	SOF+LDV	8	14	SVR12, 100.0 (40/40)
Dhiman et al. (28)	Multicenter open-label	India	G1 (9), G3 (24), G4 (2), G5 (1), unknown (21)	57/0	40 (69.3)	15.8 (12–17)	SOF+LDV;SOF+DCV ± RBV	12;24	57	SVR12, 98.3 (56/57)
Abdel Ghaffar et al. (29)	Open-label	Egypt	G4 (40)	40/0	25 (62.5)	12.27 (8–17.58)^*^	SOF+DCV	12	40	SVR12, 97.5 (39/40)
Fouad et al. (30)	Observational	Egypt	G4a (51)	35/16	32 (62.7)	14.7 (11–17.5)	SOF+LDV	12	51	SVR12, 100.0 (51/51)
El-Khayat et al. (15)	Cross-sectional	Egypt	G4 (157)	63/94	97 (62.0)	14 (12–17)	SOF+LDV	8;12	157	SVR12, 98.1 (154/157)
Nagral et al. (31)	Single-arm	India	G1 (12), G3 (5), unknown (1)	17/1	9 (50.0)	15.1 (12–17)	SOF+LDV;SOF+DCV ± RBV	12;24	18	SVR12, 88.9 (16/18)
El-Araby et al. (11)	Observational	Egypt	G4	80/20	66 (66.0)	13.8 (9–12)	SOF+LDV	12	100	SVR12, 100.0 (100/100)
Padhi (10)	Observational	India	G3 (14)	14/0	12 (85.7)	9.5 (7–13)	SOF+DCV	12	14	SVR12, 100.0 (14/14)
Mehta et al. (32)	Observational	India	G3 (10)	10/0	10 (100)	13 (11–17)	SOF+DCV	12	10	SVR12, 100.0 (10/10)
Alkaaby et al. (33)	Observational	Iraq	G1 (10), G4 (2), unknown (10)	15/7	14 (63.6)	12.5 (7–17)^*^	SOF+LDV	12	22	SVR12, 90.9 (20/22)
El-Karaksy et al. (34)	Observational	Egypt	G4 (40)	30/10	26 (65.0)	13.9 (11.5–17.5)	SOF+LDV	12	40	SVR12, 100.0 (40/40)
Yakoot et al. (35)	Multicenter, open-label	Egypt	G4 (30)	NA	17 (56.7)	12.567 (12–17)	SOF+DCV	12	30	SVR12, 96.7 (29/30)
Murray et al. (17)	Multicenter, open-label	USA	G1 (88), G3 (2), G4 (2)	72/20	84 (91.5)	9 (6–11)	SOF+LDV ± RBV	12; 24	92	SVR12, 98.9 (91/92)
Leung et al. (36)	Multicenter, open-label	USA	G1 (31), G4 (7)	25/13	13 (34.0)	15 (12–17)	OBV/PTV/R ± DSV ± RBV	12; 24	38	SVR12, 100.0 (38/38)
El-Shabrawi et al. (37)	Single-arm, multicenter	Egypt	G4 (20)	17/3	11 (55.0)	9.1 (6–12)	SOF+LDV	12	20	SVR12, 95.0 (19/20)
El-Shabrawi et al. (38)	Open-label	USA	NA	9/1	5 (50.0)	15.5 (13–17)	SOF+DCV	8	10	SVR12, 100.0 (10/10)
El-Khayat et al. (12)	Multicenter, open-label	Egypt	G4 (144)	128/16	99 (69.0)	14 (12–17)	SOF+LDV	12	144	SVR12, 98.6 (142/144)
Wirth et al. (39)	Multicenter, open-label	USA	G2 (13), G3 (39)	43/9	31 (60.0)	15 (12–17)	SOF+RBV	12; 24	52	SVR12, 98.1 (51/52)
Balistreri et al. (16)	Multicenter, open-label	USA, UK, and Australia	G1 (100)	80/20	37 (37.0)	15 (12–17)	SOF+LDV	12	100	SVR12, 98.0 (98/100)
Hashmi et al. (40)	Open-label	Pakistan	G3 (27), G1 (6), unknown (2)	35/0	22 (62.9)	10.2 (5–17)^*^	SOF+RBV	24	35	SVR12, 97.1 (30/35)
El-Shabrawi et al. (41)	Single-center	Egypt	NA	39/0	NA	16.5 (6–17)^*^	SOF+LDV/ SOF+DCV	8; 12	39	SVR12, 100.0 (39/39)
Ahmed et al. (42)	NA	Egypt	NA	NA	NA	NA (10–17)	SOF+LDV/SOF+DCV	12	40	SVR12, 100.0 (40/40)
EI-Sayed et al. (43)	Prospective pilot study	Egypt	NA	NA	NA	16 (15–17)	SOF+DCV ± RBV	12	13	SVR12, 100.0 (13/13)
El-Sayed et al. (44)	Open-label, phase 2 study	Egypt	G4 (13)	13/0	11 (85.0)	NA (12–17)	SOF+LDV	12	13	SVR12, 100.0 (13/13)
Isakov et al. (45)	NA	Egypt	NA	NA	27 (50.9)	12.5 (10–17)	SOF+LDV	8	53	SVR12, 100.0 (53/53)
Jonas et al. (46)	Open-label	NA	G1 (131), G2 (9), G3 (23), G4 (5), G6 (5)	149/26	86 (49.0)	102 (12–17); 73 (6–12)	SOF+VEL	12	102;73	SVR12, 95.0 (97/102); 92.0 (67/73)
Sheha et al. (47)	Single-center	Egypt	G4	NA	NA	NA (12–17)	SOF+LDV	12	53	SVR12, 100.0 (53/53)
Serranti et al. (48)	Multi-center study	NA	G1 (64), G3 (2), G4 (12)	NA	36 (46.2)	15.2 (12–17)	SOF+LDV	12	78	SVR12, 98.7 (77/78)

*SOF, sofosbuvir; LDV, ledipasvir; OBV, ombitasvir; PTV, paritaprevir; r, ritonavir; RBV, ribavirin; DCV, daclatasvir; DSV, dasabuvir; G, glecaprevir; P, pibrentasvir; VEL, velpatasvir.*

* *The distribution of age for subjects was undefined;*

** *Different articles form the same first author*.

The methodological quality of each study is shown in Supplementary Figures 1, 2. The quality assessment criteria according to the National Institute of Health quality assessment tools are listed in Supplementary Table 3 (https://www.nhlbi.nih.gov/health-topics/study-quality-assessment-tools). As shown in Supplementary Figure 2, most items had good level of research quality except for Q5, which was the result of patient specificity.

### Efficacy Analysis of DAAs in Adolescents With CHC

**Figure 2:**
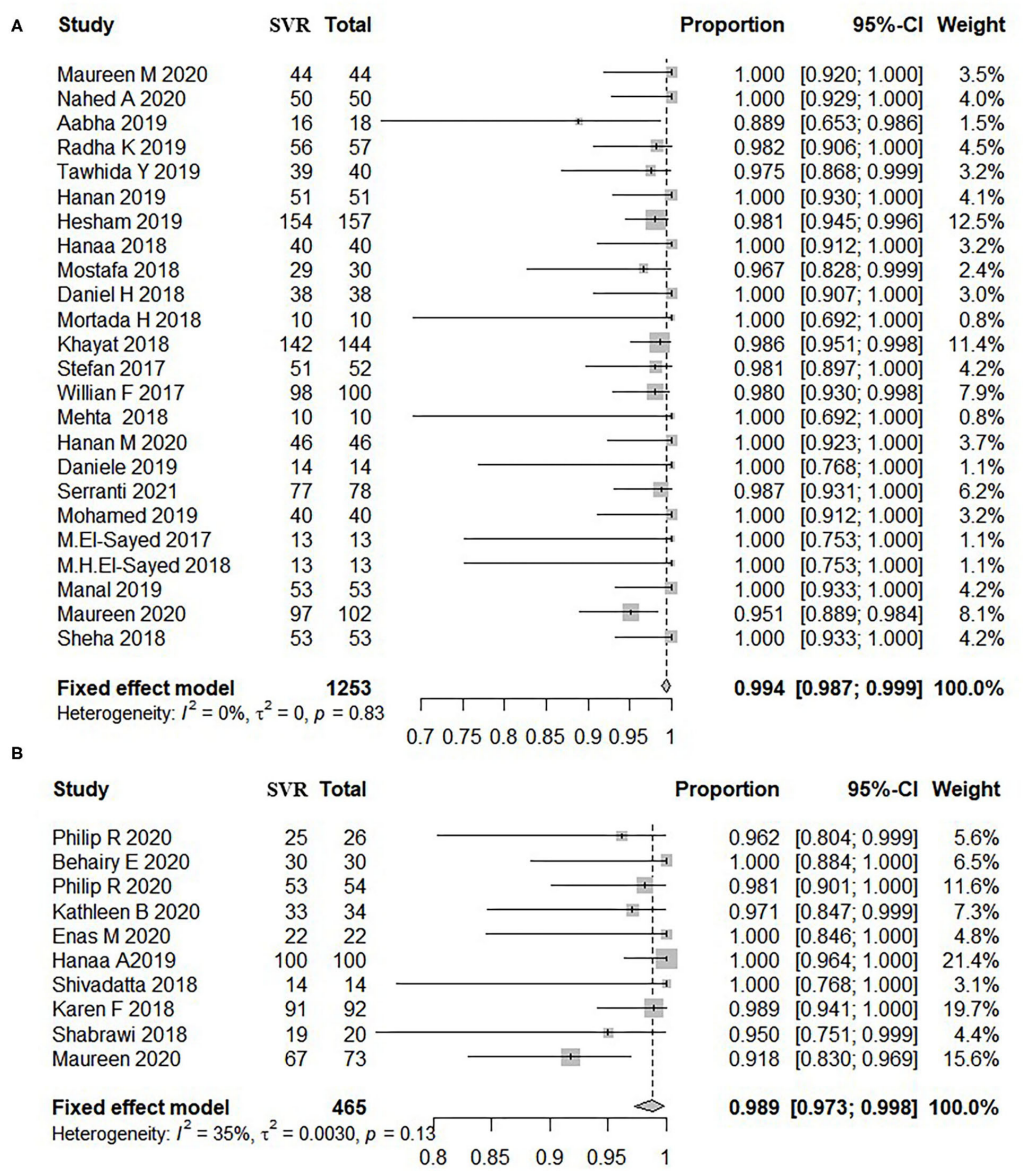
Overall rate of SVR12 in patients treated by DAAs. **(A)** Patients aged 12–17 years old. **(B)** Patients below 12 years old. The size of the square represents the weight of the study in the meta-analysis; the line width represents the 95% confidence interval of the study; the vertical line represents the “no effect line”; the diamond-shaped block represents the combined effect estimate of each study (fixed effect model or random effects model). **(A)** Patients aged 12–17 years old. **(B)** Patients below 12 years old. SVR, sustained virological response; DAAs, direct-acting antivirals; CI, confidence interval; Total, sum of patients treated by DAA.

A total of 24 studies including 1,253 adolescents patients were included for evaluating SVR12. The fixed-effect model showed that the pooled SVR12 rate was 99.4% (837/1,253, 95% CI: 98.7–99.9) (12, 15, 16, 18, 22, 23, 27–32, 34–36, 38, 39, 42–48). There was no significant heterogeneity (*I*^2^ = 0%, *P* = 0.83) (Figure 2A) or publication bias (*t* = 0.22, *P* = 0.828) (Supplementary Figure 3A) among these studies. There were three different treatment cycles of 8, 12, and 24 weeks. As shown in Table 2, the SVR12 rate was 100% (193/194, 95% CI: 98.7–100) for patients treated for 8 weeks, 99.3% (998/1,015, 95% CI: 98.4–99.9) for those treated for 12 weeks, and 100% (43/44, 95% CI: 98.9–100) for those treated for 24 weeks. The pooled SVR12 rate was 100% (95% CI: 100.0–100.0) (Supplementary Figure 4A), with litter heterogeneity among the three groups (*P* = 0.398). In addition, there were no significant differences in the pooled SVR12 rates when analyzed for the genotype, treatment history, and treatment regimen subgroups (Supplementary Figures 6A–8A).

**Table 2:** Rate of SVR12 after different durations of treatment in children and adolescents.

**Adolescents group (12–17 years)**							**Children group (<12 years)**					
**Subgroups**	**Studies (n)**	**SVR12 (*****N*** **=** **831)**		**Heterogeneity**		***P*^**b**^**	**Studies (n)**	**SVR12 (*****N*** **=** **392)**		**Heterogeneity**		***P*^**d**^**
		**Total, n/N**	**Rate% (95% CI)**	***I*^**2**^ (%)**	***P*^**a**^**			**Total, n/N**	**Rate% (95% CI)**	***I*^**2**^ (%)**	***P*^**c**^**	
By the durations of treatment						0.398						0.716
8 weeks	5	193/194	100.0 (98.7–100.0)	0	0.92		2	41/41	100.0 (95.9–100.0)	0	0.75	
12 weeks	20	998/1,015	99.3 (98.4–99.9)	0	0.78		9	410/421	98.8 (97.1–99.8)	38	0.11	
24 weeks	4	43/44	100.0 (98.9–100.0)	0	0.93		1	3/3	100.0 (50.0–100.0)	NA	NA	

*SVR, sustained virological response; CI, confidence interval; I^2^, I-square; NA, not applicable.*

a *Test of heterogeneity in adolescents group;*

b *Test for subgroup differences in adolescents group;*

c *Test of heterogeneity in children group;*

d *Test for subgroup differences in children group*.

### Safety Analysis of DAAs in Adolescents With CHC

As shown in Table 3 and Supplementary Figure 5A, the AE rate was 31, 36.1, and 41.7% among adolescents treated with DAAs for 8, 12, and 24 weeks, respectively. No significant heterogeneity was observed among three groups (31.0 vs. 36.1 vs. 41.7%, *P* = 0.918). Furthermore, the pooled AE rate for the adolescents aged 12–17 years was 34.7% (385/1,109, 95% CI: 31.9–37.6) and the SAE rate was 0.2% (95% CI: 0–0.6). The top AEs in adolescents were headache (22.6%, 206/910), abdominal pain (21.1%, 118/560), fatigue (15.5%, 129/832), nausea (15.4%, 90/585), and diarrhea (15.0%, 104/695).

**Table 3:** AEs after different treatment durations in children and adolescents.

**Adolescents group (12–17 years)**							**Children group (<12 years)**					
**Variables**	**Studies (n)**	**AE**		**Heterogeneity**		***P*^**b**^**	**Studies (n)**	**AE**		**Heterogeneity**		***P*^**d**^**
		**Total, n/N**	**Rate% (95% CI)**	***I*^**2**^ (%)**	***P*^**a**^**			**Total, n/N**	**Rate% (95% CI)**	***I*^**2**^ (%)**	***P*^**c**^**	
Durations of treatment						0.918						<0.001
8 weeks	5	55/194	31.0 (0–79.2)	98.0	<0.01		2	29/41	57.8 (0–100.0)	95.0	<0.01	
12 weeks	17	297/871	36.1 (23.3–49.9)	94.0	<0.01		8	174/385	45.1 (24.4–66.8)	94.0	<0.01	
24 weeks	4	31/44	41.7 (0–94.2)	63.0	0.05		2	37/39	98.7 (88.9–100.0)	0	1.00	

*AE, adverse event.*

a *Test of heterogeneity in adolescents group;*

b *Test for subgroup differences in adolescents group;*

c *Test of heterogeneity in children group;*

d *Test for subgroup differences in children group*.

### Efficacy Analysis of DAAs in Children With CHC

A total of nine studies including 465 pediatric patients were included for SVR12 evaluation, and the fixed-effect model showed that the pooled SVR12 rate was 98.9% (454/465, 95% CI: 97.3–99.8) (10, 11, 13, 17, 21, 24–26, 37, 46). There was little heterogeneity among these studies (*I*^2^ = 35%, *P* = 0.13) (Figure 2B), and no significant publication bias was observed as per the funnel plot (*t* = −0.68, *P* = 0.519) (Supplementary Figure 3B). Eight of these studies reported the efficacy of 12-week treatment, two studies reported the efficacy of 8-week treatment, and only one study observed the outcomes of 24-week treatment. The SVR12 rates were 100% (41/41, 95% CI: 95.9–100), 98.8% (410/421, 95% CI: 97.1–99.8), and 100% (3/3, 95% CI: 50.0–100.0) for patients treated for 8, 12, and 24 weeks, respectively (Supplementary Figure 4B). No distinct heterogeneity was observed among these groups (*P* = 0.676). Moreover, the pooled SVR12 rate for children with CHC was independent of HCV genotypes, treatment history, and treatment regimens (Supplementary Figures 6B–8B).

### Safety Analysis of DAAs in Children With CHC

As shown in Table 3, the AE rates in pediatric patients treated with DAAs for 8, 12, and 24 weeks were 57.8, 45.1, and 98.7%, respectively (Supplementary Figure 5B). Thus, the AE rate increased significantly when the treatment was continued for 24 weeks (98.7 vs. 57.8/45.1%, *P* < 0.001). The pooled AE rate was 51.6% (240/465, 95% CI: 47.0–56.2) and the SAE rate was 1.1% (5/465, 95% CI: 0.4–2.5) for children (<12 years of age), and the most common AEs were fatigue (28.4%, 80/282), headache (27.6%, 82/297), vomiting (21.1%, 51/242), cough (15.4%, 35/228), and fever (14.9%, 34/228) (Table 4).

**Table 4:** Rate of AEs, SAEs, discontinuation, and the common AEs among children and adolescents.

**Response**	**Adolescents group (12–17 years)**		**Children group (<12 years)**	
	**Total, n/N**	**Rate% (95% CI)**	**Total, n/N**	**Rate% (95% CI)**
Total AEs (not including SAEs)	385/1,109	34.7 (31.9–37.6)	240/465	51.6 (47.0–56.2)
SAEs	2/1,122	0.2 (0–0.6)	5/465	1.1 (0.4–2.5)
AEs leading to discontinuation	0/1,253	0 (0–0.3)	2/465	0.4 (0.1–1.5)
Headache	206/910	22.6 (20.0–25.5)	82/297	27.6 (22.6–33.1)
Fatigue	129/832	15.5 (13.1–18.1)	80/282	28.4 (23.2–34.0)
Diarrhea	104/695	15.0 (12.4–17.8)	–	–
Abdominal pain	118/560	21.1 (17.8–24.7)	–	–
Nausea	90/585	15.4 (12.6–18.6)	–	–
Vomiting	–	–	51/242	21.1 (16.1–26.8)
Cough	–	–	35/228	15.4 (10.9–20.7)
Fever	–	–	34/228	14.9 (10.6–20.2)

*AE, adverse event; SAE, severe adverse event*.

## Discussion

Compared to adult patients, there are significant gaps regarding the data of adolescents and children with HCV infection. Although several DAAs are effective and safe in adolescents with hepatitis C (8, 49), it is unclear whether a shorter 8-week treatment cycle would achieve similar outcomes as the 12-week or even 24-week cycles. To this end, we systematically analyzed the studies published so far on the therapeutic efficacy of DAA-containing regimens in children and adolescents with HCV infection.

Prior to the regulatory approval of DAAs for pediatric patient, the standard treatment for adolescents and children infected with HCV was 24 weeks of pegIFN and RBV for GT 2 and 3, and 48 weeks for GT 1 and 4 (50–58). This combination resulted in an SVR of around 52% in patients infected with HCV GT 1 and 4, and 89% in those infected with HCV GT 2 and 3, but was associated with significant side effects (54–56, 58). Compared to IFN-based regimens, DAAs not only are more efficient but also have fewer side effects (10–12, 15–17, 22, 24–26, 28, 29, 31, 34–39). We found that the overall SVR12 rate for the adolescents and children treated with DAAs was 99.4 and 98.9%, respectively, although the frequency of AEs was substantial (34.7 and 51.6%). Nevertheless, SAEs were rare (0.2 and 1.1%) and no adolescent patients discontinued treatment due to the AEs since most were tolerable, such as headaches (22.6%), abdominal pain (21.1%), and fatigue (15.5%). Moreover, children were more likely to experience side effects compared to teenagers (51.6 vs. 34.7%). The most common AE among children was fatigue (28.4%), most likely due to “abnormal drug taste” (24, 26). Thus, DAAs are relatively well-tolerated by both children and adolescents.

Apart from efficacy and safety, cost-effectiveness is also an important issue for any drug regimen (59). Kohli et al. (60), Latt et al. (61), and Kattakuzhy et al. (62) analyzed the outcomes of HCV treatment shorter than 12 weeks and reported ambiguous results. A recent review has shown that 8 weeks of glecaprevir/pibrentasvir (G/P) is equally effective in treatment-naive non-cirrhotic adults (63). We did not detect any significant differences between the various treatment durations in terms of efficacy in adolescents or children (*P*_*b*_ = 0.398, *P*_*d*_ = 0.716). Hesham et al. also found that 8 weeks of treatment with the SOF/LDV combination was as effective and safe as the 12-week regimen in adolescent GT4 patients (15). Similar results were reported by Mortada et al. (38). As for the treatment cycle of 24 weeks, most just appeared in RBV-based regimen in children, because SOF+RBV was also a suboptimal regimen for persons with GT 3 infection, especially if they have liver cirrhosis (8). We found that both 8-week and 12/24-week treatment courses were well-tolerated in adolescents (31 vs. 36.1%/41.7%, *P* = 0.918), whereas the AE rate at 24 weeks was greater than that at 8/12 weeks (98.7 vs. 57.8%/45.1%, *P* < 0.001) in children with CHC. This can be attributed to RBV intolerance, as well as the fact that a longer treatment duration would also increase the chances of detecting AEs that manifest late. The correlation between treatment duration and AEs needs to be studied further.

Given the underdeveloped immune system of children and the limited time for which DAAs have been administered to this group, our findings should be interpreted with caution. In addition, we only evaluated the efficacy of DAAs in terms of SVR12, and some subgroups did not have a corresponding control due to ethical reasons. Secondly, stratified analysis of SVR showed that the heterogeneity within the three treatment cycles was somewhat large, but the inter-group heterogeneity was not statistically significant. Lastly, only the FDA-approved DAAs were analyzed in the review. Therefore, the treatment outcomes of novel DAAs will have to be continuously monitored in children.

In conclusion, DAAs are overall effective and well-tolerated in adolescents and children with chronic hepatitis C. The 8-week treatment course is as effective as 12/24 weeks in both adolescents and children.

## Data Availability Statement

The original contributions presented in the study are included in the article/Supplementary Material, further inquiries can be directed to the corresponding author/s.

## Author Contributions

ZF and MY: study design and protocol, searches, title, abstract, full-text screening, data abstraction, statistical analyses, interpretation of the data, and drafting the article. ZG and CW: data verification and statistical analyses. CS and YW: statistical analyses and interpretation of the data. JL and CZ: interpretation of the data. PH, CD, and YZ: study design and protocol, interpretation of the data, and drafting the article. ZF, CD, PH, and MY: manuscript revision and question answer. All authors contributed to the article and approved the submitted version.

## Conflict of Interest

The authors declare that the research was conducted in the absence of any commercial or financial relationships that could be construed as a potential conflict of interest.
