# Supplementary material for: High SVR12 With 8-Week Course of Direct-Acting Antivirals in Adolescents and Children With Chronic Hepatitis C: A Comprehensive Analysis
Source: Front Med (Lausanne). 2021 Jun 8;8:608760. doi: 10.3389/fmed.2021.608760 (PMC8217461; doi:10.3389/fmed.2021.608760)
Supplement: Supplementary file 1 [file Data_Sheet_1.docx]

**Supplementary data**

**Abbreviations**:

**AE:** adverse event; **CHC:** chronic hepatitis C; **95% CI:** 95% confidence interval; **DAAs:** direct-acting antivirals; **DCV:** daclatasvir; **DSV:** dasabuvir; **EMA:** European Medicines Agency; **FDA:** Food and Drug Administration; **FTDA**, Freeman-Tukey double arcsine transformation. **GT:** genotype; **G/P:** glecaprevir/pibrentasvir; **HCV:** hepatitis C virus; **HCC:** hepatic carcinoma; **HBV:** hepatitis B virus; **HIV:** human immunodeficiency virus; **ITT:** intention-to-treat; **LDV:** ledipasvir; **LLOQ:** the lower limit of quantitation; **OBV:** ombitasvir; **PTV:** paritaprevir; **PRISMA:** preferred reporting items for systematic review and meta-analyses; **pegIFN:** pegylated interferon; **r:** ritonavir; **RBV:** ribavirin; **SVR:** sustained virological response; **SOF:** sofosbuvir; **SAE:** serious adverse event; **TN:** treatment-naïve; **TE:** treatment-experienced; **WHO:** World Health Organization.

**Table 1. PRISMA checklist.**

| Section/topic | # | Checklist item | Reported  on page # |
| --- | --- | --- | --- |
| TITLE | | | |
| Title | 1 | Identify the report as a systematic review, meta-analysis, or both. | 1 |
| ABSTRACT | | | |
| Structured summary | 2 | Provide a structured summary including, as applicable: background; objectives; data sources; study eligibility criteria, participants, and interventions; study appraisal and synthesis methods; results; limitations; conclusions and implications of key findings; systematic review registration number. | 2 |
| INTRODUCTION | | | |
| Rationale | 3 | Describe the rationale for the review in the context of what is already known. | 3-4 |
| Objectives | 4 | Provide an explicit statement of questions being addressed with reference to participants, interventions, comparisons, outcomes, and study design (PICOS). | 4-6 |
| METHODS | | | |
| Protocol and registration | 5 | Indicate if a review protocol exists, if and where it can be accessed (e.g., Web address), and, if available, provide registration information including registration number. | Not Applicable |
| Eligibility criteria | 6 | Specify study characteristics (e.g., PICOS, length of follow-up) and report characteristics (e.g., years considered, language, publication status) used as criteria for eligibility, giving rationale. | 5-7 |
| Information sources | 7 | Describe all information sources (e.g., databases with dates of coverage, contact with study authors to identify additional studies) in the search and date last searched. | 4-5 |
| Search | 8 | Present full electronic search strategy for at least one database, including any limits used, such that it could be repeated. | 5;Supplementary table 2 |
| Study selection | 9 | State the process for selecting studies (i.e., screening, eligibility, included in systematic review, and, if applicable, included in the meta-analysis). | 5-7;Figure 1 |
| Data collection process | 10 | Describe method of data extraction from reports (e.g., piloted forms, independently, in duplicate) and any processes for obtaining and confirming data from investigators. | 7 |
| Data items | 11 | List and define all variables for which data were sought (e.g., PICOS, funding sources) and any assumptions and simplifications made. | 6-7 |
| Risk of bias in individual studies | 12 | Describe methods used for assessing risk of bias of individual studies (including specification of whether this was done at the study or outcome level), and how this information is to be used in any data synthesis. | 7 |
| Summary measures | 13 | State the principal summary measures (e.g., risk ratio, difference in means). | 6 |
| Synthesis of results | 14 | Describe the methods of handling data and combining results of studies, if done, including measures of consistency  (e.g., I^2^) for each meta-analysis. | 7 |
| Risk of bias across studies | 15 | Specify any assessment of risk of bias that may affect the cumulative evidence (e.g., publication bias, selective reporting within studies). | 7 |
| Additional analyses | 16 | Describe methods of additional analyses (e.g., sensitivity or subgroup analyses, meta-regression), if done, indicating which were pre-specified. | 8 |
| RESULTS | | | |
| Study selection | 17 | Give numbers of studies screened, assessed for eligibility, and included in the review, with reasons for exclusions at each stage, ideally with a flow diagram. | 8; Figure 1 |
| Study characteristics | 18 | For each study, present characteristics for which data were extracted (e.g., study size, PICOS, follow-up period) and provide the citations. | 8-9;Table 1 |
| Risk of bias within studies | 19 | Present data on risk of bias of each study and, if available, any outcome level assessment (see item 12). | 9;Supplementary figure 1 |
| Results of individual studies | 20 | For all outcomes considered (benefits or harms), present, for each study: (a) simple summary data for each intervention group (b) effect estimates and confidence intervals, ideally with a forest plot. | 8-11;Figure 2 ; Table2 |
| Synthesis of results | 21 | Present results of each meta-analysis done, including confidence intervals and measures of consistency. | 9-13; Table2 |
| Risk of bias across studies | 22 | Present results of any assessment of risk of bias across studies (see Item 15). | 7;Supplementary figure 2 |
| Additional analysis | 23 | Give results of additional analyses, if done (e.g., sensitivity or subgroup analyses, meta-regression [see Item 16]). | Supplementary figure 4-8 |
| DISCUSSION | | | |
| Summary of evidence | 24 | Summarize the main findings including the strength of evidence for each main outcome; consider their relevance to key groups (e.g., healthcare providers, users, and policy makers). | 11-14 |
| Limitations | 25 | Discuss limitations at study and outcome level (e.g., risk of bias), and at review-level (e.g., incomplete retrieval of identified research, reporting bias). | 13-14; Supplementary figure 2 |
| Conclusions | 26 | Provide a general interpretation of the results in the context of other evidence, and implications for future research. | 13-14 |
| FUNDING | | | |
| Funding | 27 | Describe sources of funding for the systematic review and other support (e.g., supply of data); role of funders for the systematic review. | 14 |

**Table 2. Full search strategy.**

| **Database** | **Number of identified records** |
| --- | --- |
| **PubMed** | **741** |
| ((((((HCV) OR hepatitis C virus) OR CHC) OR hepatitis C) AND (DAA or direct-acting antiviral or Sofosbuvir or Dasabuvir or Daclatasvir or Ledipasvir or Ombitasvir or Elbasvir or Velpatasvir or Boceprevir or Telaprevir or Simeprevir or Asunaprevir or Paritaprevir or Grazoprevir) AND (children or child or teenager or kid or adolescent or youngster or juvenile) |  |
| **Cochrane library** | **188** |
| (HCV or hepatitis C virus or hepatitis C or CHC):ti,ab,kw AND (DAA or direct-acting antiviral or Sofosbuvir or Dasabuvir or Daclatasvir or Ledipasvir or Ombitasvir or Elbasvir or Velpatasvir or Boceprevir or Telaprevir or Simeprevir or Asunaprevir or Paritaprevir or Grazoprevir):ti,ab,kw AND (children or child or teenager or kid or adolescent or youngster or juvenile):ti,ab,kw |  |
| **Web of Science** | **1344** |
| Subject: (HCV or hepatitis C virus or hepatitis C or CHC) AND Subject: (DAA or direct-acting antiviral or Sofosbuvir or Dasabuvir or Daclatasvir or Ledipasvir or Ombitasvir or Elbasvir or Velpatasvir or Boceprevir or Telaprevir or Simeprevir or Asunaprevir or Paritaprevir or Grazoprevir) AND Subject: (children or child or teenager or kid or adolescent or youngster or juvenile) |  |

**Table 3. Questions for Quality Assessment for non-randomized-controlled trials according to the National Institute of Health quality assessment tool for “before-after (pre-post) studies with no control group”** (<https://www.nhlbi.nih.gov/health-topics/study-quality-assessment-tools>)

| 1. Was the study question or objective clearly stated? |
| --- |
| 2. Were eligibility/selection criteria for the study population prespecified and clearly described? |
| 3. Were the participants in the study representative of those who would be eligible for the test/service/intervention in the general or clinical population of interest? |
| 4. Were all eligible participants that met the prespecified entry criteria enrolled? |
| 5. Was the sample size sufficiently large to provide confidence in the findings? |
| 6. Was the test/service/intervention clearly described and delivered consistently across the study population? |
| 7. Were the outcome measures prespecified, clearly defined, valid, reliable, and assessed consistently across all study participants? |
| 8. Were the people assessing the outcomes blinded to the participants' exposures/interventions? |
| 9. Was the loss to follow-up after baseline 20% or less? Were those lost to follow-up accounted for in the analysis? |
| 10. Did the statistical methods examine changes in outcome measures from before to after the intervention? Were statistical tests done that provided *p* values for the pre-to-post changes? |
| 11. Were outcome measures of interest taken multiple times before the intervention and multiple times after the intervention (i.e., did they use an interrupted time-series design)? |
| 12. If the intervention was conducted at a group level (e.g., a whole hospital, a community, etc.) did the statistical analysis take into account the use of individual-level data to determine effects at the group level? |


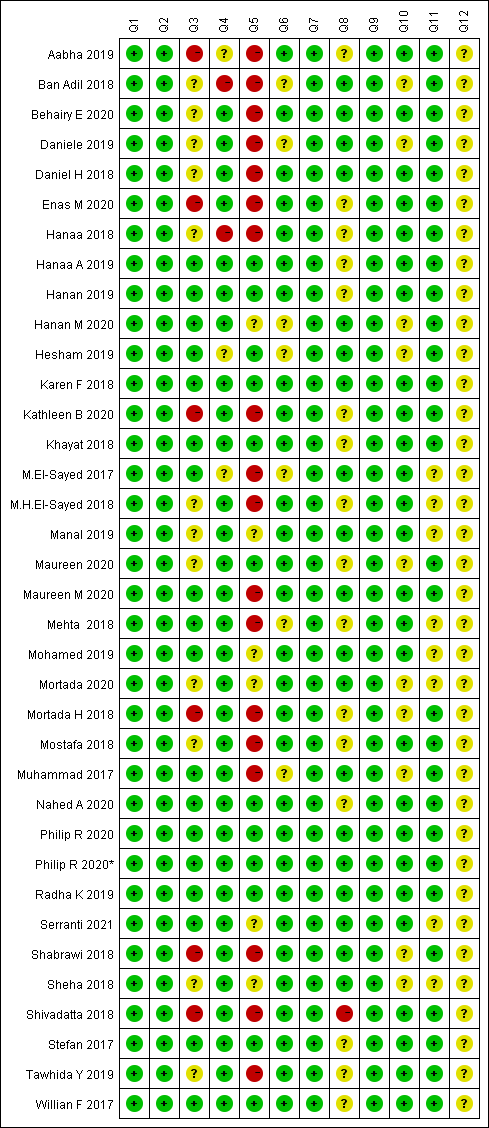


**Figure 1. Assessment about each risk of bias item for each included study.**
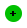
Low bias risk;
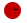
High bias risk;
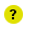
Unclear risk of bias. ^*^ Different articles form the same first author.


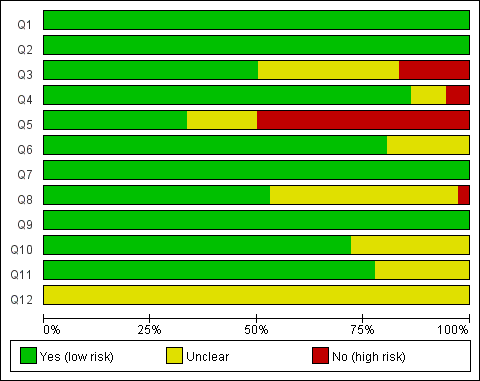


**Figure 2. Assessment of each risk of bias item presented as percentages across all included studies.**


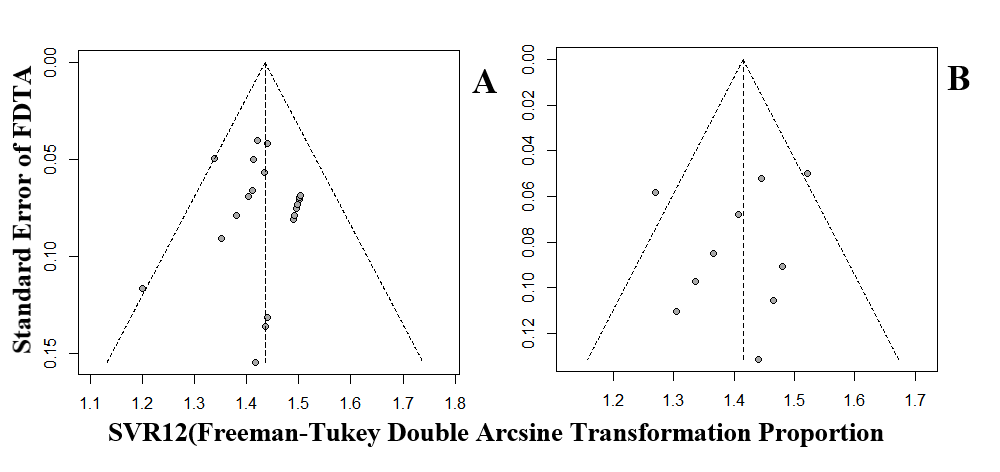


**Figure 3. Funnel plot analysis of studies reporting efficacy of DAA therapy.** Dots refer to included publications. (A) Adolescents aged 12-17 years old; (B) Children aged below 12 years old. DAAs: direct-acting antivirals; FTDA, Freeman-Tukey double arcsine transformation.


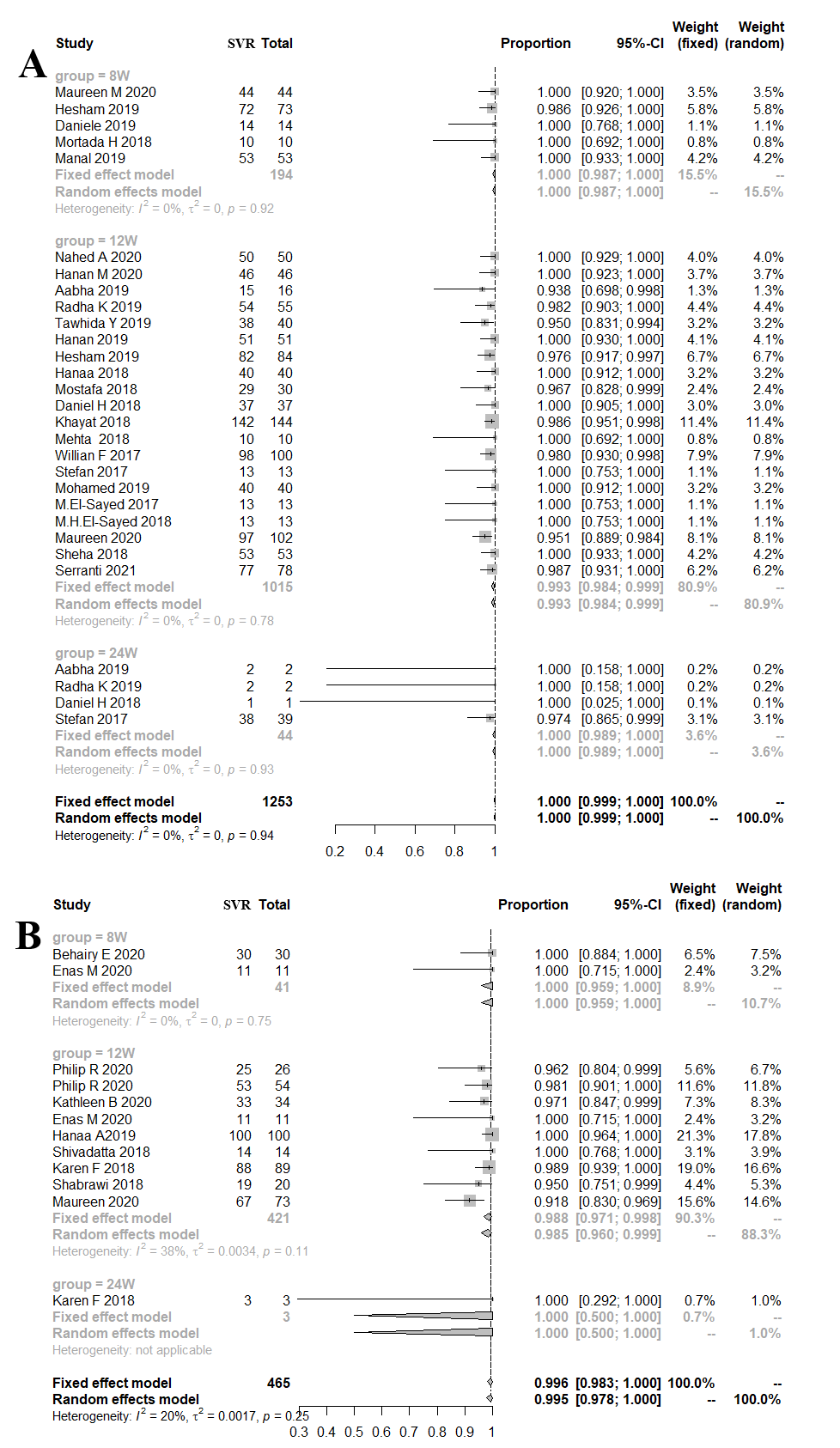


**Figure 4. SVR12 of different treatment durations among children and adolescents.** (A) Patients aged 12-17 years old; (B) Patients below 12 years old; SVR: sustained virological response.


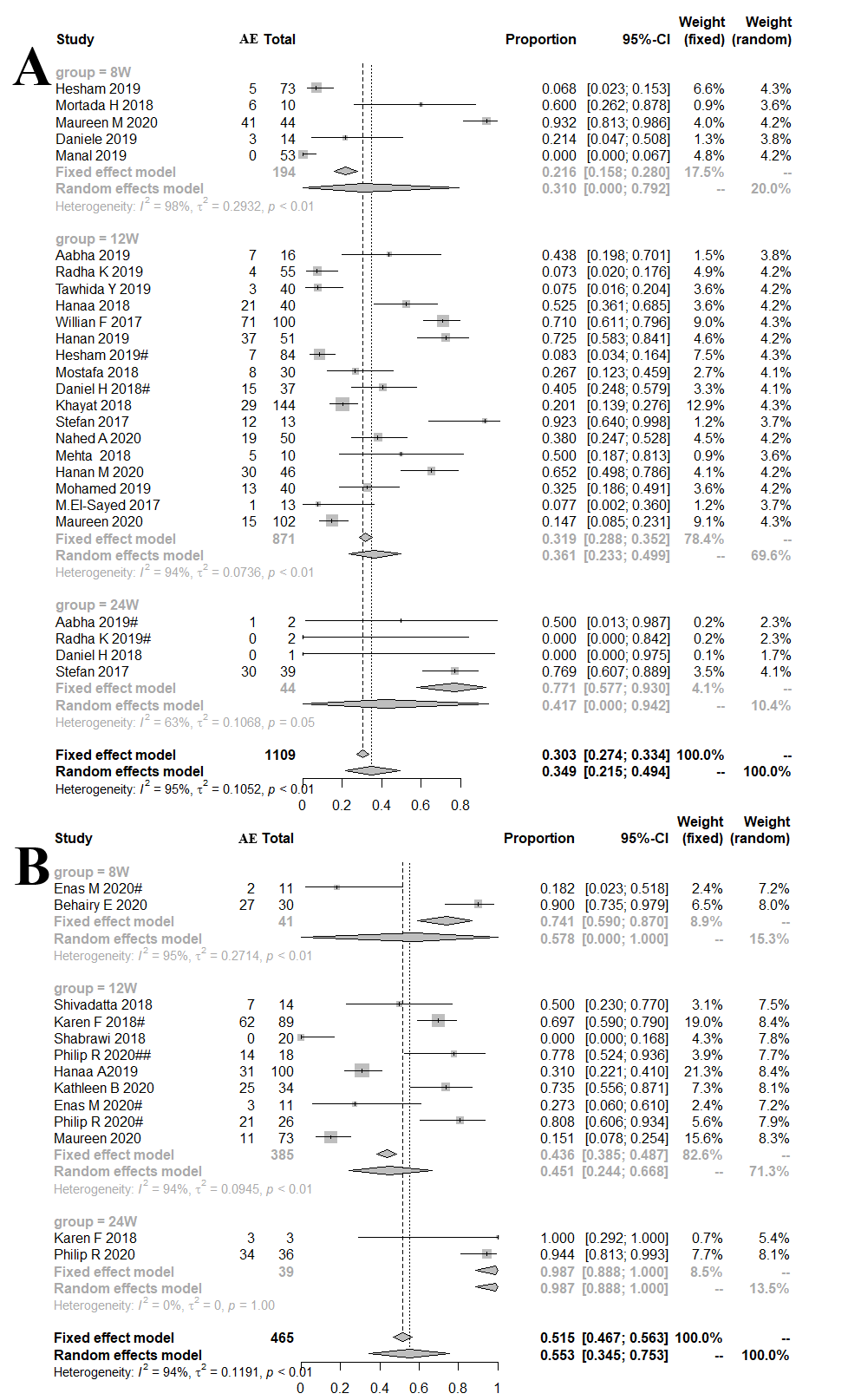


**Figure 5. AEs rates after different treatment durations in children and adolescents.** (A) Patients aged 12-17 years old; (B) Patients below 12 years old; AE: adverse event.

**
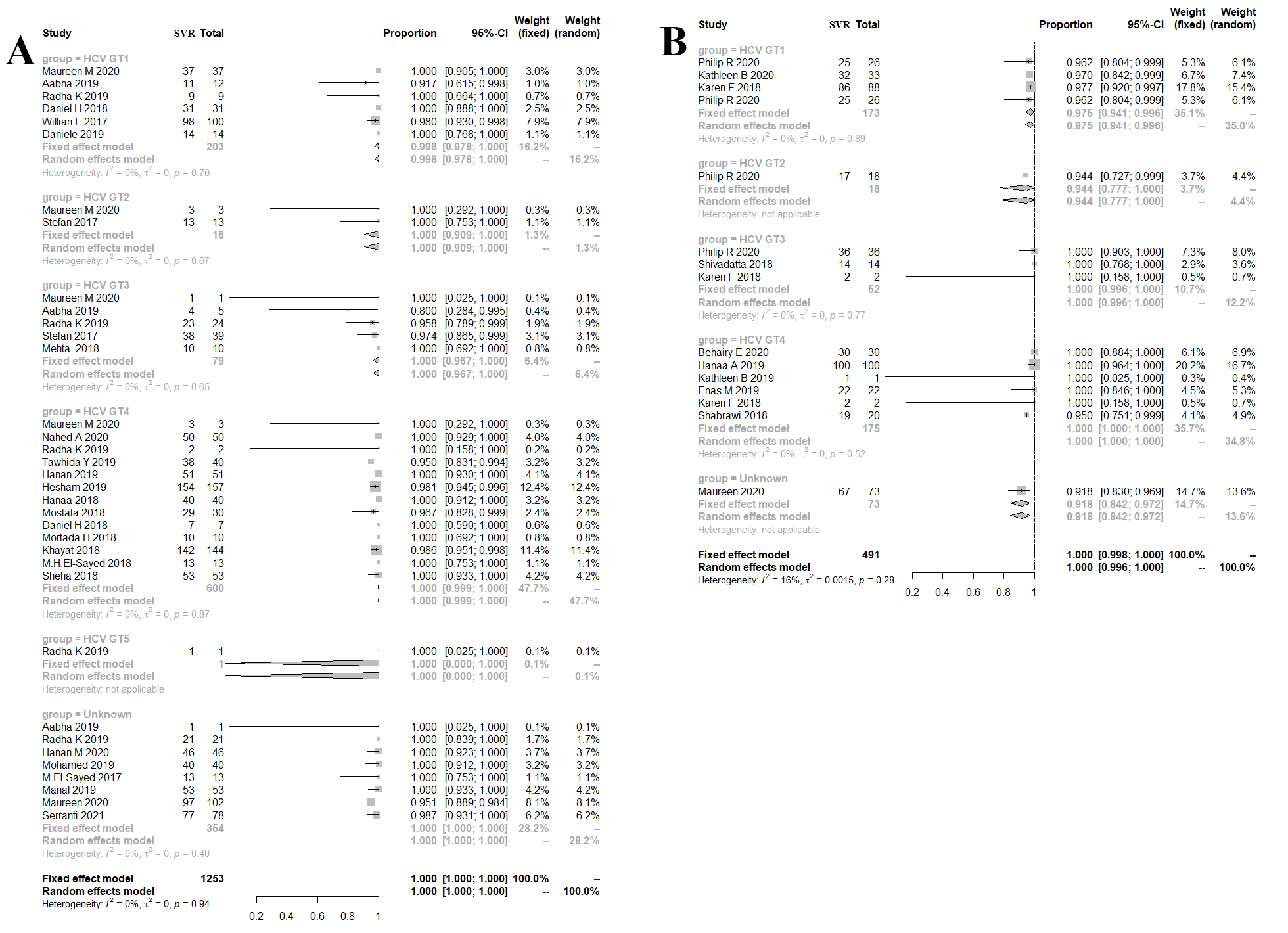
**

**Figure 6. SVR12 in different genotype subgroups.** (A) Patients aged 12-17 years old; (B) Patients below 12 years old; SVR: sustained virological response.


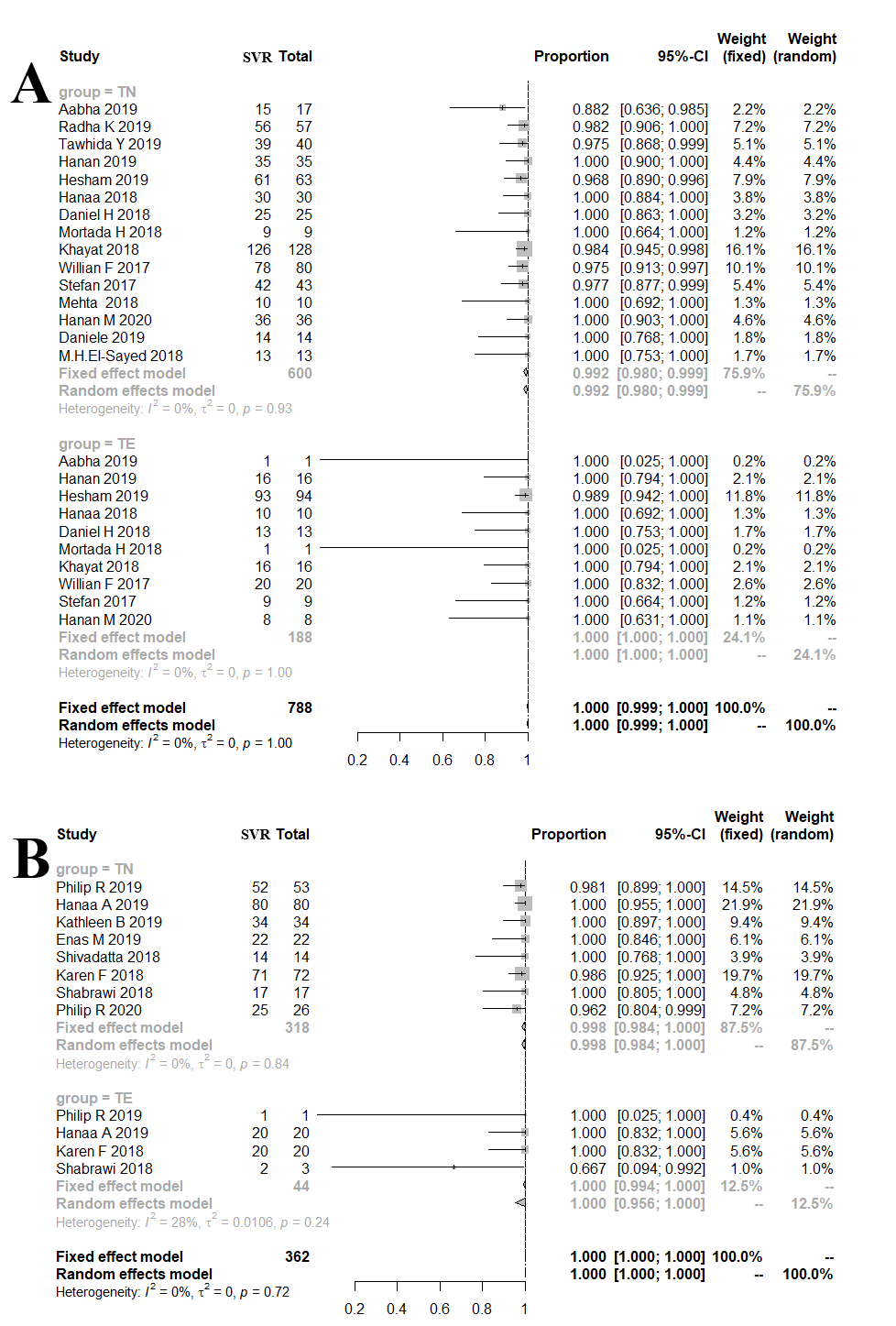


**Figure 7. SVR12 in subgroups stratified by history of treatment.** (A) Patients aged 12-17 years old; (B) Patients below 12 years old; SVR: sustained virological response.


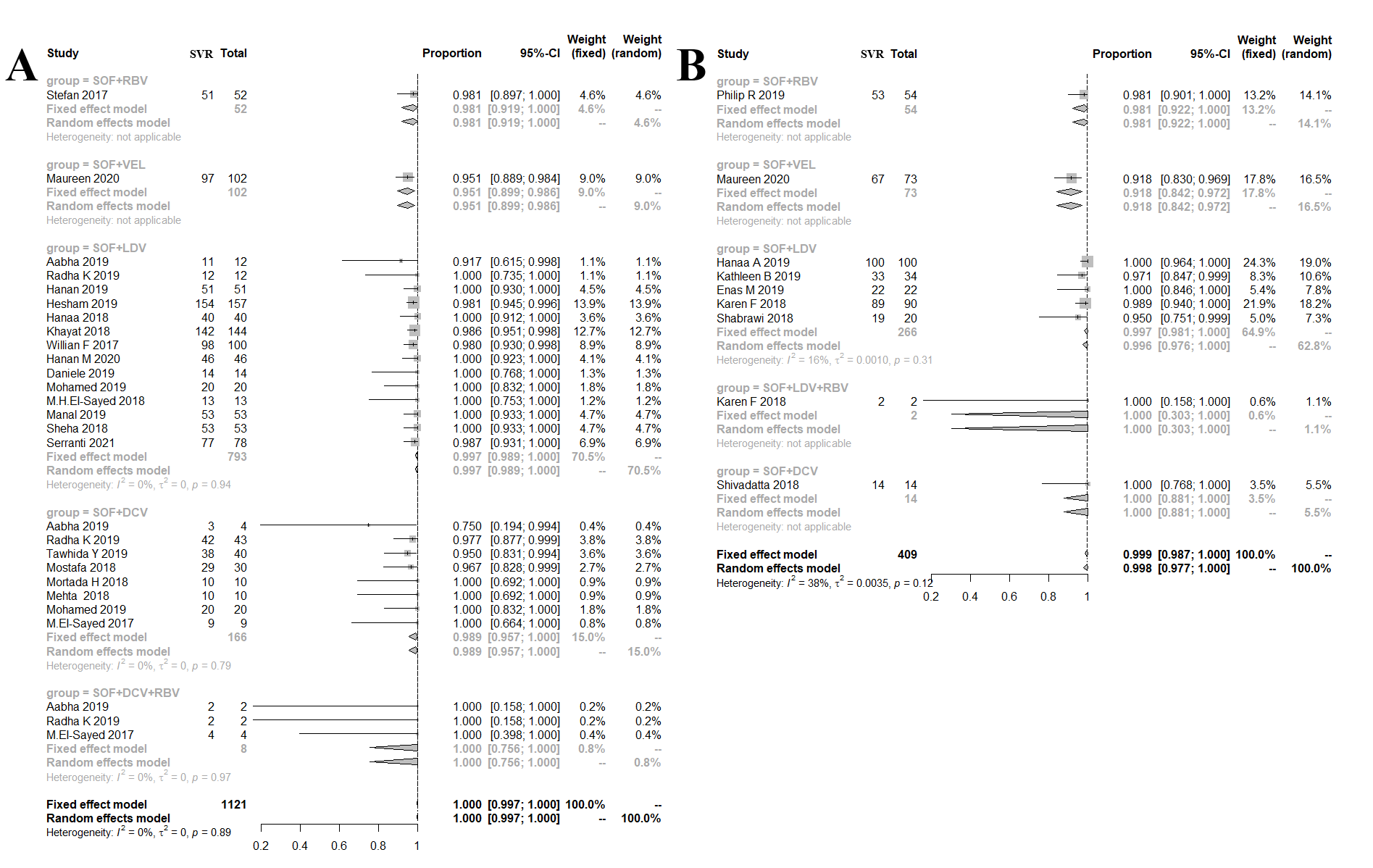


**Figure 8. SVR12 in different treatment regimen subgroups.** (A) Patients aged 12-17 years old; (B) Patients below 12 years old; SVR: sustained virological response.
